# Quality of Clinical Information in Pregnancy Pharmacovigilance Data Sources—A Contribution of the ConcePTION Project

**DOI:** 10.1002/pds.70182

**Published:** 2025-06-25

**Authors:** Yrea R. J. van Rijt‐Weetink, Jip van Gendt, Toine C. G. Egberts, Florence P. A. M. van Hunsel, David J. Lewis, Laura M. Yates, Ursula Winterfeld, Eugène P. van Puijenbroek

**Affiliations:** ^1^ Groningen Research Institute of Pharmacy, PharmacoTherapy, ‐Epidemiology and ‐Economics, University of Groningen Groningen the Netherlands; ^2^ Division of Pharmacoepidemiology and Clinical Pharmacology, Department of Pharmaceutical Sciences Faculty of Science, Utrecht University Utrecht the Netherlands; ^3^ Department of Clinical Pharmacy University Medical Centre Utrecht Utrecht the Netherlands; ^4^ Pharmacovigilance Centre Lareb, 's‐Hertogenbosch 's‐Hertogenbosch the Netherlands; ^5^ Global Drug Development, Novartis Pharma GmbH Wehr Germany; ^6^ School of Life and Medical Sciences, University of Hertfordshire Hatfield UK; ^7^ KRISP, University of Kwazulu‐Natal Durban South Africa; ^8^ Northern Genetics Service, Newcastle‐upon‐Tyne Hospitals NHS Foundation Trust Newcastle upon Tyne UK; ^9^ Swiss Teratogen Information Service and Clinical Pharmacology Service, Centre Hospitalier Universitaire Vaudois (CHUV) and University of Lausanne Lausanne Switzerland

**Keywords:** pharmacovigilance, pregnancy, quality

## Abstract

**Purpose:**

Good documentation of adverse events related to medicines is essential for the assessment of safety signals. Information on the clinical quality of primary pregnancy safety data sources is lacking. The objective of this study was to assess the differences in clinical quality of various sources of primary pregnancy pharmacovigilance (PV) data.

**Methods:**

Fifty reports of exposures to medicines during pregnancy were collected from: spontaneous and literature reports from EudraVigilance, European Network of Teratology Information Services (ENTIS), the Dutch Pregnancy Drug Register, enhanced PV programmes (EPV), and patient support programmes (PSP). Reports were standardized and anonymized, after which their clinical quality was assessed. Mean scores per source were compared using ANOVA (analysis of variance test).

**Results:**

Mean clinical quality scores were 89.0% (SD 10.1%) for the Dutch Pregnancy Drug Register, 77.1% (SD 13.3%) for TIS, 64.7% (SD 20.5%) for EPVs, 49.5% (SD 16.2%) for PSPs, 40.9% (SD 21.6%) for spontaneous reports, and 38.6% (SD 18.0%) for literature reports. All were statistically significantly different (*p* ≤ 0.05) except for spontaneous versus literature reports (mean difference 2.2%, *p* = 0.99) and spontaneous reports versus reports from PSPs (−8.6%, *p* = 0.14).

**Conclusions:**

For data sources specifically designed for pregnancy data collection, the clinical quality of information generally outweighed sources designed to capture general safety information. EPV methods showed better scores for clinical quality compared to spontaneous reporting data for pregnancy PV.

**Summary**

- Good clinical documentation of adverse events related to exposure to medicinal products is important for the reliable assessment of potential safety signals.
- Case assessment aims to evaluate causal strength, best supported by high‐quality clinical documentation, often captured more effectively in narratives than coded data.
- For data sources specifically designed for pregnancy pharmacovigilance, the clinical quality of information surpasses data sources designed to capture information on the safety of medicinal products in general.
- Enhanced pharmacovigilance methods show better scores for clinical quality compared to general spontaneous reporting data collection for pregnancy pharmacovigilance.
- Generic systems are less suited for the specific purpose of pregnancy data collection, but may be improved by adopting approaches used in more specialized pharmacovigilance programmes.

## 1 Introduction

While acknowledging the need for reliable information on potential risks associated with maternal and foetal exposure to medicinal products during pregnancy, practical challenges arise in effectively collecting and disseminating this information to healthcare providers and consumers. Since pregnant women are generally excluded from clinical studies for ethical reasons [1], information regarding adverse events associated with the use of medicinal products during pregnancy usually has to be obtained from daily life experiences [2]. For this purpose, various sources of primary data are available.

Current adverse event monitoring systems rely on spontaneous reporting by health care professionals (HCPs) or patients concerning the presence or absence of an adverse outcome. These reports are submitted to national pharmacovigilance (PV) centres and the marketing authorization holders (MAHs) of the products who undertake an assessment of likely causality. Set components of the reports are forwarded to regulatory authorities such as the European Medicines Agency (EMA) and, on a global level, the World Health Organization (WHO) collaborating centre—the Uppsala Monitoring Centre (UMC) [3, 4]. These reports, also referred to as Individual Case Safety Reports (ICSRs), adhere to a predefined, internationally harmonized ICH(E2B)‐R3 structure. This structure is a technical standard developed by the International Council for Harmonisation of Technical Requirements for Pharmaceuticals for Human Use (ICH). It enables the electronic transmission of ICSRs in a standardized format between parties involved in pharmacovigilance. Mandatory information includes amongst others reactions/event, drug(s) information, patient characteristics, and a narrative case summary and further information [5].

In addition to spontaneous reporting, MAHs are responsible for identifying, submitting and reporting case reports described in medical literature that may contain relevant information of pregnancy exposure to their licenced products as ICSRs in the same way as voluntary reports. A third source is information that originates from organized data collection systems, for example, patient support programs, pharmacoepidemiologic studies and non‐interventional studies that can be submitted as ICSRs. This type of information is referred to as ‘solicited reports’ [6]. In addition, some MAHs have initiated so‐called enhanced pharmacovigilance programmes, where spontaneously reported adverse outcomes after medicinal product exposure during pregnancy are enriched using structured follow‐up focused on exposure during pregnancy at set intervals [7]. Primary data can also be collected in dedicated pregnancy registries, in which pregnant women are followed over time by means of questionnaires [8, 9]. Finally, Teratology Information Services (TIS) collaborate through the European Network of Teratology Information Services (ENTIS) or the Organization of Teratology Information Specialists (OTIS), to improve knowledge on the safety of use of medicinal products during pregnancy and lactation [10, 11]. They counsel HCPs and sometimes consumers regarding exposure to medicinal products during pregnancy and lactation, and collect information on the pregnancy outcomes when women have been exposed to these products during pregnancy.

The quality of the aforementioned data sources may vary, which may impact their utility. Clinically well‐documented reports are more likely to support a reliable assessment of potential safety signals [12, 13]. A tool for the assessment of the clinical quality of information in pregnancy PV case reports has previously been developed and validated [14, 15]. This standardized method is designed to quantify the suitability of information for the purpose of assessing the causal relationship between exposure to a medicinal product and the presence or absence of an adverse outcome during pregnancy and can be considered to be a measure of the clinical quality. It considers the relevance of elements of information for assessing a specific event and the actual presence or absence of these information elements. As an example, information about the timing of exposure in relation to the gestational age is relevant for assessing the causal relationship between a suspected medicinal product and a reported congenital disorder. If this information is absent, the causality assessment will be less reliable, and therefore the quality of the report is lower. Conversely, the presence or absence of information regarding the mode of delivery in a report describing a potential association between a medicinal product and gestational diabetes does not influence the assessment. Hence, it does not impact the quality of the report, making this element of information irrelevant in this context.

We hypothesized that not all data sources contribute equally to the assessment of pregnancy PV safety signals. Some sources are probably more suitable for this purpose than others. However, a formal assessment of the clinical quality of the various primary pregnancy PV data sources is currently lacking. The objective of this study was to assess the differences in clinical quality of various sources of primary pregnancy PV data.

## 2 Methods

### 2.1 Setting and Design

This study is part of Work Package 2 of the IMI funded ConcePTION project, in which national PV centres, MAHs, and TIS centres collaborated in the optimization of the collection, analysis, and interpretation of reported pregnancy PV data [16].

In this study, we aimed to include 50 reports from each of the following data sources:

1. Spontaneous reports from health care professionals or consumers captured in the EudraVigilance database (EMA) [4],
2. Case reports published in medical literature captured in the EudraVigilance database [4],
3. Patient support programme reports from Novartis [17],
4. Enhanced PV programme reports from Argus Safety (Novartis) [7],
5. Pregnancy registry reports from the Dutch Pregnancy Drug Register (Lareb) [9], and
6. Teratology information services reports from ENTIS, including TIS centres in the UK, Switzerland, and two centres in Israel [10].

Case reports had to be completed, closed (lost to follow‐up) or (if relevant) submitted to EudraVigilance between January 1st, 2020 and December 31st, 2021. It was therefore possible that initial intake or data collection started before this timeframe. Both prospective (reported prior the pregnancy outcome being known) and retrospective case reports (reported after pregnancy outcome had become available) could be included, provided that an association to be assessed was present, consisting of a medicinal product and an adverse pregnancy event, adverse child outcome, or reported healthy pregnancy.

Reports related to only one or more of the following situations were excluded: medicinal product exposure during breast feeding, paternal drug exposure, non‐pregnancy‐related complications, delivery or post‐partum complications of the mother, or when the report concerned a COVID‐19 vaccine. The latter exclusion criterion was applicable to prevent the large number of reports on COVID vaccines over recent years from making the sample less representative. From all eligible reports available in a data source, up to 50 reports were randomly selected.

### 2.2 Data Assessment

Case reports were blinded for the data source, by reformatting the data to a standardized format and extracted into a specifically designed spreadsheet. Any details that may have revealed the origin of the case reports and privacy sensitive details were replaced with equivalent anonymized information (e.g., geographical references, non‐English terminology, contact information). If multiple adverse outcomes in a single case report were reported that fulfilled the in‐ and exclusion criteria, one of the outcomes was chosen at random. For assessing the clinical quality, case reports were presented in a random order. Each case report was assessed based on the relevance and presence of various elements of information. This method has been recently validated for the assessment of the clinical quality of information in pregnancy pharmacovigilance data sources. A full description of the method and validation has been published elsewhere [14, 15].

In this study, the assessment of the relevance of the information elements was performed in two steps. In the first step, one researcher (EvP) marked which elements of information were generally considered to be relevant for reported adverse outcomes that were included more than once in the dataset. This assessment was discussed in a focus group discussion with pregnancy PV experts from the pharmacovigilance centre Lareb, the national PV centre in the Netherlands. Thereafter, this general assessment for the selected reported outcomes was applied to all individual case reports to facilitate the identification of relevant elements of information. Nevertheless, the relevance of elements of information was checked for exceptions independently by two researchers (YvRW and JvG) for all case reports. These two researchers also conducted the second step, individually evaluating whether the relevant information elements were sufficiently available to assess causality. Differences were discussed until agreement was reached. In case of no agreement, a third assessor (EvP) decided on the relevance and presence.

An overview of the various elements of information, divided into main categories, can be found in Table 1. It should be noted that, depending on the nature of reported events and medicinal products, not all information elements listed under a specific category had to be present. As an example, in respect to information related to the child, in case of a low Apgar score at birth, information on breastfeeding is not relevant. It was up to the assessor to determine which of the listed elements of information are considered relevant. Therefore, it was important that the assessor had the required teratology experience to assess the relevance and presence of the elements of information. Per individual case report, the number of information elements that were relevant and the number of information elements that were relevant and present were counted, after which the clinical quality score was calculated by the number of relevant and present elements of information divided by the number of relevant elements of information times 100. All reports with a score of < 45% are classified as poor clinical quality, 45%–65% are classified as intermediate clinical quality, and ≥ 65% are classified as excellent clinical quality [15]. The information element medicinal product‐event combination is always relevant and present.

**TABLE 1:** Method for the assessment of clinical quality of case reports in pregnancy related pharmacovigilance data based on presence and relevance of information [14, 15].

Category	Elements of information	Relevance	Presence
Association	Medicinal product—event combination		
Event details	Information to validate the diagnosis of the event (e.g., test results) Timing of occurrence or detection of the event Chronologic evolution of the event, possibly in relation to the exposure		
Medicinal product exposure details	Administration information of medicinal product (e.g., dose, route) Timing of exposure of medicinal product in relation to timing of gestation Indication for use of medicinal product Other exposures (including additional information on e.g., dose, route, timing, and indication)		
Maternal factors	Medical history and concurrent disorders of the mother Maternal demographics (e.g., age, weight) Lifestyle and risk factors (e.g., smoking, alcohol)		
Pregnancy	Previous pregnancies Pregnancy‐related complications of current pregnancy Prenatal testing		
Labour	Labour onset Mode of delivery Delivery complications		
Child	Gestational age at birth Apgar score (1–5–10 min) Breastfeeding Medical information of neonate (e.g., weight, diagnoses that are not the reported event)		

*Note:* Clinical quality = # present and relevant elements of information/# Relevant elements of information × 100.

### 2.3 Data Analysis

All case reports were unblinded for the data source, and the mean and median clinical quality scores with standard deviation and interquartile range were calculated per data source. Using ANOVA and Tukey–Kramer post hoc tests (R stats Package, version 4.4.0), it was determined for which data sources the mean clinical quality was statistically significantly different. Additionally, for every individual element of information, the percentage of completeness (i.e., how often the information element was reported when it was marked as relevant) was calculated per data source. The percentage of completeness was calculated by the following formula: number of reports in which the information element was both present and relevant/the number of reports in which the information element was relevant × 100. Results were visualized in a boxplot (R ggpubr package, version 0.6.0) and heatmap (MS Excel). All elements of information that were assessed as being relevant in less than five case reports were excluded. Statistical calculations were done using Rstudio version 4.4.0.

## 3 Results

We intended to include 50 reports from all data sources, but for the spontaneous reports, patient support programme reports, and the enhanced PV programme reports a total number of respectively 47, 45, and 28 from the provided 50 fulfilled the inclusion criteria. All data sources initially provided 50 cases. During data cleaning, it became apparent that some of these cases were unfortunately unusable. However, the timeline of the research project did not allow for an additional data extraction. For this reason, some data sources have fewer than the originally intended 50 cases.

**FIGURE 1:**
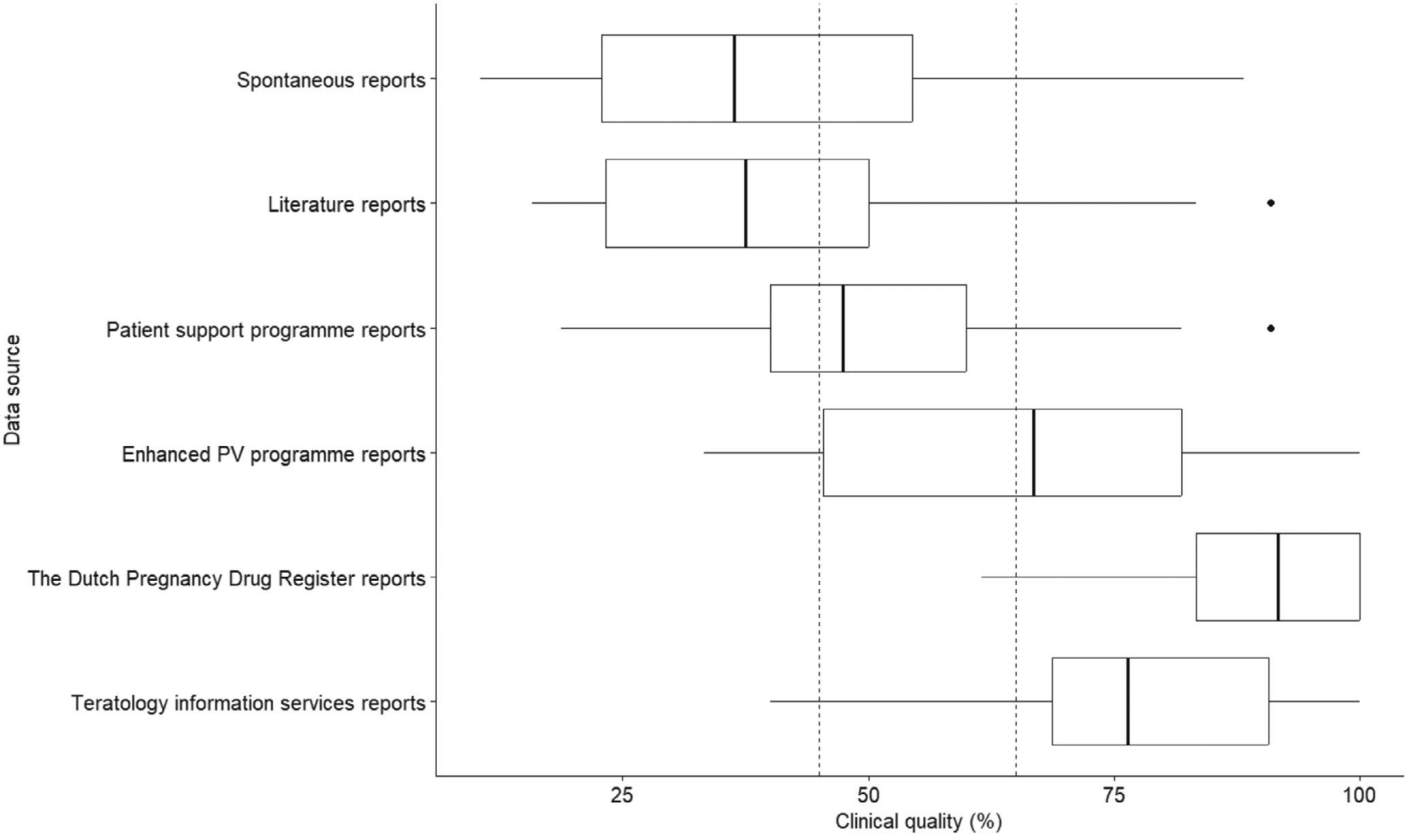
Boxplots of clinical quality per data source, expressed as percentage of present and relevant over relevant elements of information. The boxplots consist of the median quality score (bold line), interquartile range (box), minimum to maximum quality score (whiskers) and if applicable outliers (dots). Vertical lines at the cut‐off values between categories are shown; < 45% poor clinical quality, 45%–65% intermediate clinical quality, and ≥ 65% excellent clinical quality. PV, pharmacovigilance.

In total, 270 case reports were included, ranging from the 28 of the enhanced PV programme reports to 50 case reports per source from TIS, literature cases, and the Dutch Pregnancy Drug Register (Table 2). The mean clinical quality score ranged from 39% (standard deviation [SD]: 18%) for literature reports to 89% (SD: 10%) for reports from the Dutch Pregnancy Drug Register. Of all ICSRs, less than 20% of case reports were categorized as excellent clinical quality. For literature reports, this percentage was 6% (*n* = 3), while for spontaneous reports and patient support programme reports, it was slightly higher at 12.8% (*n* = 6) and 15.6% (*n* = 7) respectively. Both TIS reports and reports from the Dutch Pregnancy Drug Register were categorized as excellent in 90% or more of the reports: 90% (*n* = 45) of TIS reports and 98% (*n* = 49) of reports from the Dutch Pregnancy Drug Register. Figure 1 depicts the distribution of the clinical quality of the data sources in boxplots.

**TABLE 2:** Characteristics of the case reports per data source. Columns poor, intermediate and excellent show the number of case reports classified in that respective category.

Data source	Number of case reports	Mean quality score (SD)	Median quality score (IQR)	Poor	Intermediate	Excellent
Spontaneous reports	47	41 (22)	36 (23–54)	29 (61.7%)	12 (25.5%)	6 (12.8%)
Literature reports	50	39 (18)	38 (23–50)	30 (60%)	17 (34%)	3 (6%)
Patient support Programme reports	45	50 (16)	47 (40–60)	15 (33.3%)	23 (51.1%)	7 (15.6%)
Enhanced PV programme reports	28	65 (21)	67 (45–82)	6 (21.4%)	8 (28.6%)	14 (50%)
The Dutch pregnancy drug register reports	50	89 (10)	92 (83–100)	0 (0%)	1 (2%)	49 (98%)
Teratology information services reports	50	77 (13)	76 (69–91)	2 (4%)	3 (6%)	45 (90%)
Total	270	60 (26)	61 (40–83)	82 (30.4%)	64 (23.7%)	124 (45.9%)

Comparing the mean clinical quality of the different data sources using ANOVA showed a statistically significant result (*F*‐value 73, *p* < 0.001). Subsequent Tukey–Kramer post hoc tests are presented in Table 3. All comparisons of the mean clinical quality between two data sources were statistically significantly different, except for the comparison between literature reports and spontaneous reports (mean difference − 2.2%, *p* = 0.99) and between patient support programme reports and spontaneous reports (8.6%, *p* = 0.14).

**TABLE 3:** Tukey–Kramer post hoc test results for the comparison of mean clinical quality between data sources in the rows compared to the sources mentioned in the columns.

	Spontaneous reports	Literature reports	Patient support programme reports	Enhanced PV programme reports	The Dutch pregnancy drug register reports	Teratology information services reports
Spontaneous reports						
Literature reports	−2.2% (*p* = 0.99)					
Patient support programme reports	8.6% (*p* = 0.14)	10.9% (*p* = 0.02)				
Enhanced PV programme reports	23.9% (*p* < 0.001)	26.1% (*p* < 0.001)	15.3% (*p* < 0.01)			
The Dutch Pregnancy Drug Register reports	48.1% (*p* < 0.001)	50.3% (*p* < 0.001)	39.5% (*p* < 0.001)	24.2% (*p* < 0.001)		
Teratology information services reports	36.2% (*p* < 0.001)	38.5% (*p* < 0.001)	27.6% (*p* < 0.001)	12.3% (*p* = 0.02)	−11.9% (*p* < 0.01)	

Abbreviation: PV, pharmacovigilance.

Elements of information related to the event details (validation, timing and evolution) were reported poorly in all data sources (Table 4). The only exception was timing of the event, which was reported in 68.2% of case reports for which it was considered relevant in the Dutch Pregnancy Drug Register. For elements of information related to the medicinal product exposure details, all information elements were excellently reported for the enhanced PV programme reports and the Dutch Pregnancy Drug Register reports, and all but one for TIS reports (64.3% for indication). For ICSRs all but one information element was reported poorly or intermediately (66.7% for indication in literature reports). Also noteworthy is that elements of information related to labour were generally reported poorly, with the exception of labour onset and mode of delivery for reports from the Dutch Pregnancy Drug Register (both 100%) and mode of delivery for reports from TIS (57.1%).

**TABLE 4:** Percentages of completeness for individual elements of information. All elements of information that were assessed as relevant in less than five case reports were excluded.

		Spontaneous reports (*n* = 47)	Literature reports (*n* = 50)	Patient support programme reports (*n* = 45)	Enhanced PV programme reports (*n* = 28)	The Dutch Pregnancy Drug Register reports (*n* = 50)	Teratology information services reports (*n* = 50)
Association	Product‐event combination	100.0 (47/47)	100.0 (50/50)	100.0 (45/45)	100.0 (28/28)	100.0 (50/50)	100.0 (50/50)
Event details	Validation	25.0 (9/36)	21.4 (9/42)	22.2 (2/9)	(0/1)	16.7 (4/24)	13.3 (2/15)
	Timing (event)	25.0 (9/36)	40.0 (16/40)	39.1 (9/23)	37.5 (3/8)	68.2 (16/22)	18.8 (3/16)
	Evolution (event)	28.1 (9/32)	27.3 (12/44)	33.3 (7/21)	(2/4)	29.2 (7/24)	5.9 (1/17)
Medicinal product exposure details	Administration information	45.5 (20/44)	33.3 (14/42)	82.2 (37/45)	82.1 (23/28)	89.2 (33/37)	70.2 (33/47)
	Timing (exposure)	34.0 (16/47)	35.7 (15/42)	53.3 (24/45)	78.6 (22/28)	77.6 (38/49)	79.6 (39/49)
	Indication	50.0 (20/40)	66.7 (22/33)	(1/1)	(0/1)	96.6 (28/29)	64.3 (27/42)
	Other exposures	55.3 (26/47)	54.8 (23/42)	31.1 (14/45)	75.0 (21/28)	100.0 (49/49)	81.6 (40/49)
Maternal factors	Medical history	27.7 (13/47)	26.0 (13/50)	40.0 (18/45)	42.9 (12/28)	100.0 (50/50)	80.0 (40/50)
	Maternal demographics	32.6 (15/46)	11.1 (5/45)	47.7 (21/44)	73.1 (19/26)	100.0 (50/50)	89.8 (44/49)
	Lifestyle and risk factors	9.1 (4/44)	15.4 (6/39)	11.4 (5/44)	32.1 (9/28)	100.0 (49/49)	87.8 (43/49)
Pregnancy	Previous pregnancies	24.4 (10/41)	16.3 (7/43)	9.7 (3/31)	22.2 (2/9)	100.0 (20/20)	94.4 (17/18)
	Pregnancy complications	17.6 (6/34)	11.4 (4/35)	12.5 (4/32)	23.8 (5/21)	76.1 (35/46)	47.8 (22/46)
	Prenatal testing	16.7 (2/12)	5.3 (1/19)	21.4 (3/14)	(2/3)	(3/3)	(1/2)
Labour	Labour onset	21.4 (3/14)	0 (0/10)	11.8 (2/17)	(0/4)	100.0 (15/15)	12.5 (1/8)
	Mode of delivery	0 (0/6)	(0/4)	(1/3)	(0/0)	100.0 (11/11)	57.1 (4/7)
	Delivery complications	6.7 (1/15)	0 (0/24)	0 (0/7)	(0/1)	35.7 (5/14)	25.0 (2/8)
Child	Gestational age at birth	32.1 (9/28)	45.5 (15/33)	64.5 (20/31)	80.0 (16/20)	100.0 (41/41)	97.8 (45/46)
	Apgar score	23.8 (5/21)	13.3 (4/30)	0 (0/9)	(0/0)	61.1 (11/18)	53.3 (8/15)
	Breastfeeding	14.3 (1/7)	14.3 (1/7)	(2/3)	(0/0)	100.0 (7/7)	100.0 (8/8)
	Medical information child	52.0 (13/25)	41.4 (12/29)	65.5 (19/29)	54.5 (12/22)	100.0 (37/37)	93.5 (43/46)

Abbreviation: PV, pharmacovigilance.

## 4 Discussion

### 4.1 General Findings

Improving surveillance of drug safety during pregnancy is an important topic of pharmacovigilance (PV) [18]. Information regarding the safety of medicinal product use during pregnancy is drawn from various primary data sources. Information within data sources designed specifically for collecting information on exposure during pregnancy was generally of superior clinical quality. As an example, our results show that teratology information services and pregnancy registries are generally better suited for pregnancy PV compared to pregnancy exposure data collected through systems that make use of the ICH‐E2B(R3) data structure [5] that is used for exchanging ICSRs between parties involved in drug safety monitoring, that is, patient support programmes, enhanced PV programmes, spontaneous reports, and literature reports. This is not surprising, as registries or data sources specifically designed to map medicinal product exposures during pregnancy will collect targeted information that takes into account that the effects of a medication taken in pregnancy may impact the foetus or exposed child in a variety of ways, in some cases only becoming evident years after birth. On the other hand, primary sources such as literature reports, spontaneous reports, enhanced PV programmes, and patient support programmes are bound to the ICH‐E2B(R3) data model, developed originally for enabling data exchange of ICSRs relating to exposure‐adverse event pairs outside of the pregnancy setting. While the majority of ICSRs collected worldwide concern a patient's exposure to one or more drugs, pregnancy PV involves the exposure of both mother and child, with adverse reactions potentially affecting both. The data fields of the ICH‐E2B(R3) model therefore often inadequately reflect this reality. Although all reported content and all narratives and free‐text fields of the ICSRs were included in the analyses, it is possible that not all relevant information was initially captured and reported. Additionally, in some databases, it is not always possible to discern whether the reported adverse event related to the mother or child. However, in enhanced PV programmes, specific information is requested by means of targeted follow‐up, which can be included as free text in the ICSR. This will enhance its utility and can provide useful information for pregnancy PV as is reflected in a higher quality score.

The lack of dedicated fields in the ICH‐E2B format to capture pregnancy‐related information is recognized by EMA, as the guideline on Good Pharmacovigilance Practices III lists additional information elements that could be collected but are not captured in the ICH‐E2B message format. This information should be provided in the case narrative if possible [19]. To improve the utility of ICH‐E2B compatible ICSRs for pregnancy PV, in future updates of this data model, special attention should be paid to allow for storage of relevant information, like the elements of information used in our study or core data elements defined by Richardson et al. [20, 21].

### 4.2 What Is Known in Literature?

Our study shows that spontaneous reporting systems generally score lower on clinical quality for pregnancy data. In 2017, a web‐based questionnaire to 172 countries was used to ask PV centres about their current activities concerning the surveillance of drug safety during pregnancy. The response rate was 40%, and the majority of PV centres did not have additional questions specifically about the pregnancy present on their ADR reporting form. Only a limited number of PV centres actually performed pregnancy‐specific signal detection activities on their data [18]. Although pregnancy registries score higher on clinical quality and have several strengths over other surveillance methods, it is widely recognized that they also have a number of limitations, such as low levels of enrolment and loss to follow‐up. These two issues, combined with a low frequency of the exposure and outcome of interest, limit the statistical power and validity of pregnancy exposure registries [22]. In addition to the primary data sources that were included in this study, a large number of other data sources are now being used or explored for drug safety in pregnancy research [22, 23, 24]. These include population‐based surveillance registers that rely on linked data sets, healthcare databases, and purpose‐built data sources such as case–control surveillance systems [22]. Given the fact that different data sources all have their own strengths and limitations, a combined approach using a range of data sources could enhance signal detection and signal strengthening for pregnancy PV [22]. A robust understanding of the relatability of each of these systems will be fundamental to synthesizing the outputs into an overall assessment of safety or risk.

### 4.3 Methodological Considerations

The method applied in this study is a standardized approach designed to evaluate the suitability and presence of information for assessing the causal relationship between exposure to a medicinal product and the occurrence or absence of an adverse outcome during pregnancy. This method can be considered a measure of clinical quality. Having sufficient information is essential to form an opinion on the strength of the causal relationship. However, the nature of the event being assessed, the circumstances, and the methods used by assessors (e.g., algorithmic or global introspection) may vary [25]. As a result, it is not feasible to establish universal cut‐off values for causality assessments. Generally, higher clinical quality tends to provide more reliable information needed for causality assessment. However, the outcome of the causality assessment itself does not have to correlate with the quality of information.

Studies on the quality of ICSRs typically concentrate on the completeness of information, without considering the content of the information itself. As an example, the VigiGrade system developed by the Uppsala Monitoring Centre (UMC) estimates the amount of clinically relevant information in an Individual Case Safety Report (ICSR) within VigiBase, the UMC's database [3]. This includes factors such as the presence or absence of information like time‐to‐onset, indication, outcome, and the existence of free text in the reports. However, the mere presence of information does not guarantee its utility for PV purposes. More recent studies have focused on making distinctions between cases with a high or low clinical utility based on automated algorithms, which could aid in the prioritization of ICSRs for clinical review [26, 27, 28, 29]. These methods, however, were not specifically designed for pregnancy PV. The method applied in our study has been specifically designed and validated to analyse the content itself and is also applicable to datasets that may not be ICH‐E2B(R3) compatible.

Adverse event reports can include a range of pregnancy outcomes, for example, pregnancy loss, occurrence of congenital anomalies, foetal, neonatal or maternal complications, complications in the child, and finally a normal pregnancy and birth with a healthy mother and neonate(s). In this study, all reports from a specific source were combined for comparison. However, it is conceivable that differences in the clinical quality between these sources may exist for various clinical scenarios. Unfortunately, we were not able to stratify for the various scenarios due to the limited power of the study. Likewise, previous studies have shown that there are some differences in elements of information which are reported by HCPs and patients/consumers [30]. In this study, we did not specifically investigate differences in the quality of information between reporters.

The nature of the clinical outcomes from different sources varies to a certain extent. A characteristic of diverse pregnancy pharmacovigilance sources is that they encompass certain types of outcomes more frequently than others. For example, events reported to a Patient Support Program for a specific drug will differ from those reported to a general pregnancy registry. Adjusting the analysis for the nature of the outcomes would therefore not provide a correct representation of reality. In pharmacovigilance, it is crucial to use multiple sources to obtain a comprehensive and balanced view of safety. The aim of the article is precisely to highlight these differences, knowing that other outcomes will be reported. Although PV reports usually focus on comparative rates of adverse events determined using disproportionality analyses, pregnancy PV relies heavily on capturing exposure to the product without a negative outcome. To gain understanding of its safety, information from different primary data sources is essential to obtain a comprehensive view of the number of exposures that also involve a negative outcome. For this reason, these reports without a negative outcome were also included in the development and validation of the method used to assess the clinical quality [14, 15], and in the current comparison. Among the various data sources, reports without a negative outcome were reported as follows: Spontaneous reports (2 out of 47), Patient support programme (10 out of 45), Enhanced PV programme (16 out of 28), the Dutch Pregnancy Drug Register (23 out of 50), and the Teratology information services (30 out of 50). The literature reports did not include any reports without a negative outcome.

The information sources used in this study may not be representative for primary data collections in general. First of all, data used was from MAHs and regulatory agencies within the EU only. Additionally, participants of the ConcePTION project are specifically interested in the topic of medicinal product exposure during pregnancy, which may have led to an increase of clinical quality of the data collected before the start of this study specifically, compared to non‐participants. It should be noted that the design and scope of pregnancy registries and enhanced PV programmes may differ greatly between the ones included in this study and other registries and programmes. The same registry and enhanced PV programme as were used for the validation of the method were used in this study. A pregnancy register may refer to a register focused on a specific exposure, a specific disease, or both. The registry used in this study is a pregnancy exposure registry, though it may also include women who were not exposed to a medicinal product. Although six different primary data sources were used, it cannot be assumed that other primary data sources within each category are of similar nature and quality. Finally, both the patient support programme reports and the enhanced PV programme include both data from only one MAH, which may limit the generalizability of the results.

To limit changes in the quality of reports over time, the inclusion period was restricted to a two‐year timeframe. The inclusion period in this study coincided with the early years of the COVID pandemic, in which large numbers of reports related to COVID vaccines were reported. For this reason, reports concerning COVID‐19 vaccines were excluded. However, it remains unclear whether societal changes during the COVID period influenced the nature, number, and quality of reports related to maternal exposure to medications.

### 4.4 Practical Implications

In pregnancy pharmacovigilance, it is essential to obtain a comprehensive understanding of reported events. The aim of case assessment is typically to evaluate the strength of the causal relationship. The value of a case therefore increases as the clinical quality of its documentation improves. This information cannot always be captured in coded data and is sometimes better expressed in narrative text fields. The approach described in this study takes the coded information as well as the information in the narratives into account. Since clinical quality is critical for assessing causality, this information should be an integral part of the causality evaluation, which currently is not the case.

This study highlights areas for improvement in existing and future data collection systems. Generic systems, such as the ICH E2B‐R3 format, are less suited for the specific purpose of pregnancy data collection but can be improved by adopting approaches used in more specialized systems such as enhanced PV programmes. Additionally, lessons can be learned from the methods of data collection of specific systems such as TIS and pregnancy registers.

## 5 Conclusion

For data sources specifically designed for pregnancy data collection (e.g., the Dutch Pregnancy Drug Register and TIS centres), the clinical quality of information generally outweighs data sources designed to capture information on the safety of medicinal products in general (e.g., enhanced PV programmes, patient support programmes, spontaneous reports, and literature reports). Additionally, enhanced PV programme methods show better scores for clinical quality compared to general spontaneous reporting data collection for pregnancy pharmacovigilance. The identified gaps in quality of clinical information may offer opportunities for targeted interventions to improve the overall quality of pregnancy pharmacovigilance.

### 5.1 Plain Language Summary

This study aimed to assess the quality of various sources of pharmacovigilance (PV) data related to adverse events during pregnancy. Fifty reports of exposures to medicines during pregnancy were collected from different sources. The reports were standardized and anonymized, and their clinical quality was assessed. The results showed that the Dutch Pregnancy Drug Register had the highest mean clinical quality score, followed by reports from Teratology Information Services (TIS). Enhanced PV programs (EPV) and Patient support programmes (PSPs) had higher scores compared to spontaneous reports and literature reports. Except for the latter two sources, differences in scores were statistically significant. The study concluded that sources specifically designed for pregnancy data collection generally had higher clinical quality scores compared to sources designed for general safety information. EPV methods showed better scores for clinical quality compared to spontaneous reporting data for pregnancy pharmacovigilance.

## Author Contributions

The concept of this study was designed within the ConcePTION project and led by Yrea R.J. van Rijt‐Weetink, David J. Lewis, Laura M. Yates, Ursula Winterfeld, and Eugène P. van Puijenbroek. The protocol was written by Yrea R.J. van Rijt‐Weetink, Jip van Gendt, and Eugène P. van Puijenbroek. The data collection and analysis were performed by Yrea R.J. van Rijt‐Weetink, Jip van Gendt, and Eugène P. van Puijenbroek, and were regularly discussed with Toine C.G. Egberts, Florence P.A.M. van Hunsel, David J. Lewis, Laura M. Yates, and Ursula Winterfeld. All authors contributed to the article and approved the submitted version.

## Ethics Statement

The authors have nothing to report.

## Consent

The authors have nothing to report.

## Conflicts of Interest

David J. Lewis is a full‐time employee of Novartis Pharma AG and holds shares in Alcon, GlaxoSmithKline, Novartis, and Sandoz. Laura M. Yates provided consultancy for Sanofi Genzyme South Africa on two occasions in 2021 relating to genetic testing in Gaucher's Disease (April 2021, Advisory Board presentation) and a conference lecture on genetic testing in cardiac clinics (September 2021). The other authors declare no conflicts of interest.

## Data Availability

The codes and datasets for this manuscript are not publicly available because of the data protection policy of the Pharmacovigilance Centre Lareb. Requests to access the datasets should be directed to the corresponding author and will be granted on reasonable request.
